# Farm working experience could reduce late-life dependency duration among Japanese older adults: The Yamanashi Healthy–Active Life Expectancy cohort study based on the STROBE guidelines

**DOI:** 10.1097/MD.0000000000022248

**Published:** 2020-09-18

**Authors:** Kayo Haruyama, Hiroshi Yokomichi, Zentaro Yamagata

**Affiliations:** a Department of Occupational Therapy, Iryo Sosei University, 5-5-1 Chuodai Iino, Iwaki City, Fukushima; b Department of Health Sciences, Graduate School of Medicine, University of Yamanashi, Shimokato, Chuo City, Yamanashi, Japan.

**Keywords:** agriculture, dependency, elderly, life expectancy, nursing care

## Abstract

With the advance of medical care, the duration of dependency on nursing care in later life has increased worldwide. There is a question of whether farm work could extend or shorten the dependency duration. We investigated the association between farm work experience and the duration of dependency on nursing support or care in late life.

We randomly selected 600 adults aged ≥65 years, who were independent and not hospitalized, as part of the Yamanashi Healthy–Active Life Expectancy cohort and followed them for 13 years. We defined the duration of dependency as the time from reception of long-term care insurance benefits to death, and we adjusted for multiple covariates.

We analyzed data from 225 adults (139 men and 86 women) who died during the follow-up period. Ninety four had received long-term care benefits. Mean age was 79.6 years (standard deviation [SD]: 6.3) in individuals with farm work experience and 80.1 years (SD: 7.2) in individuals without farm work experience. The estimated duration of dependency on long-term care was 1.3 years (standard error [SE]: 0.4) in individuals with farm work experience vs 2.1 years (SE: 0.5) in individuals without farm work experience (*P* = .01). The estimated duration of dependency in individuals with farm work experience and without farm work experience was 0.4 years (SE: 0.5) vs 1.3 years (SE: 0.6) in men respectively (*P* = .03) and 1.6 years (SE: 0.9) vs 2.4 years (SE: 0.9) in women, respectively (*P* = .16). The sensitivity analysis yielded an estimated duration of 1.2 years (SE: 0.5) in those with farm work experience and 2.3 years (SE: 0.5) in those without farm work experience (*P* = .004).

Individuals with farm work experience required less long-term care prior to death, suggesting that agricultural and physical activities promote health. Policymakers focusing on preventing the need for nursing care in older populations could consider promoting farming or gardening.

## Abstract 2

Supplemental Digital Content is available in the text

## 1 Introduction

Improving medical care in later life has been identified as a global public health priority^[1]^ to promote older adults independence in activities of daily living.^[2]^ Several studies suggest, however, that the duration of dependency on nursing care in later life has increased worldwide.^[3,4]^

Some studies have compared health indices in farm workers and the general population, and reported that farm workers have a lower cardiovascular risk^[5]^ and lower rates of metabolic syndrome.^[6]^ In city settings, the activity of gardening reportedly leads to better health and higher well-being scores in both younger and older adults.^[7]^ Agricultural activities in later life may also protect against dementia.^[8,9]^

However, these findings do not necessarily translate to a longer lifespan in farm workers. Compared to the general population, farmers have a higher risk of vehicular accidents,^[10]^ mental disorders,^[11]^ suicide,^[12,13]^ and orthopedic diseases,^[12]^ and they may be chronically exposed to pesticides.^[14,15]^ In contrast, farmers are typically physically active,^[5–7]^ have access to fresh vegetables,^[5–7,16]^ and are less likely to smoke.^[17–19]^ There are currently no studies that have investigated the association between farm work and the duration of dependency on nursing care. We studied the association in a Japanese cohort of older adults.

## 2 Methods

### 2.1 Study design, setting, and participants

The setting was Yamanashi Prefecture, Japan, which is adjacent to Tokyo Prefecture^[20]^ and has approximately 800,000 residents and a large aging population.^[21]^ The proportion of residents aged 65 years or older was 29.8% in 2017, which is comparable to the nationwide proportion of 27.7%.^[22]^ Yamanashi Prefecture reportedly has the most daytime sunlight in Japan, contributing to the proliferation of agricultural activities.^[21]^

Participants were enrolled in 2 stages. We first selected 1800 non-institutionalized older adults from the prefecture on a probability basis for a cross-sectional study in 2002,^[23]^ with a response rate of 93.3% (1680 participants). In 2003, 600 of the participants were randomly selected for more detailed questionnaires and consented to participate in the Yamanashi Healthy–Active Life Expectancy (Y–HALE) cohort study. At baseline, none of the participants were recipients of long-term care insurance (LTCI) reimbursements, and therefore were not registered as requiring nursing care.^[23,24]^ Since 2004, these participants have been followed and monitored for activities of daily living, reception of LTCI reimbursements, and death.

### 2.2 Measurements

At baseline, we shared the questionnaire by mail.^[25]^ It is known that cerebrovascular diseases, fracture, musculoskeletal diseases, and dementia require nursing support or care.^[26,27]^ Therefore, in addition to demographic variables (sex and age), we obtained information about smoking status (never, former, or current) and alcohol consumption (once a week or less) as lifestyle habits that affect the state of health, and education background (graduation from secondary or lower school/ high school or higher), income from employment as social status. Furthermore, since there is a possibility that participants will not apply for LTCI if they have a family member, we collected information on whether they were living alone. Farm work requires physical activity, which may lead to healthy worker effects. Therefore, we also collected information about their exercise habits and about their participation in social activities, such as volunteering, or any local events.

We used the duration of dependency as the primary outcome. In Japan, the long-term care insurance benefit is ranked from 1 to 2 with respect to the need for support and from 1 to 5 for care needs.^[24]^ In this study, we defined duration of dependency as the time from the start of receiving any LTCI benefits to death. Participants who died without receiving benefits were considered to have a null year for the duration of dependency. We received notification of participant deaths from their families and local newspaper announcements.^[24]^ We also collected information about the outcome of registration for nursing care or support. Each year, in October, we mailed questionnaires to participants asking whether they had registered as requiring nursing care, the type of care required, and the date of registration for LTCI.^[24]^ We obtained information on long-term care outcomes from participants geriatric care managers.^[28]^ Consequently, the main analysis included data from 225 (of 253) participants who died between 2004 and 2017. We excluded 28 participants who had missing data regarding the date of death, information about LTCI, or baseline survey questionnaires.

### 2.3 Statistical analyses

We divided participants into 2 groups by farm work experience. We used *t*-tests to compare continuous variables and Chi-Squared tests to compare proportions. We used an analysis of covariance to estimate the primary outcome of the duration of dependency including adjusting for potential confounders. We also stratified the data by sex. Only one female participant was a current smoker, as Japanese women typically do not smoke cigarettes^[29–31]^; therefore, we eliminated smoking status as an adjusted covariate when analyzing womens data.

### 2.4 Sensitivity analysis

We excluded participants who died within 2 years of the beginning of follow-up (2004) from the sensitivity analysis to eliminate inverse causality because healthy individuals were more likely to be physically able to engage in farm work. We performed another sensitivity analysis, adjusting for exercise habits and social activities.

All analyses were conducted using SPSS version 25 (IBM SPSS, Armonk, NY, US). All reported *P* values were two-sided; *P* < .05 was considered significant.

### 2.5 Ethics statement

The ethics committee of the School of Medicine, University of Yamanashi approved this study (no. 17152) and it was conducted in accordance with the ethical guidelines and regulations of the Declaration of Helsinki. Participants provided written, informed consent and were informed that they could drop out of the study with a written request or telephone call if they decided to discontinue participation. Participants also had the opportunity to comment on the study results and could convey their views about the study by completing a questionnaire and mailing it to the study group.

## 3 Results

We followed participants for 13 years until September 2017. Table 1 lists the characteristics of participants who died during the follow-up period (n = 225). Mean age at baseline was 79.8 ± 6.6 years, and 139 of the participants (61.8%) were men. In total, 94 participants (41.8%) received insurance benefits for nursing support or care during the study period. Mean lifespan among participants was 87.3 ± 7.1 years. Supplementary Table 1, http://links.lww.com/MD/E867 lists more details about the cohort.

**Table 1:**
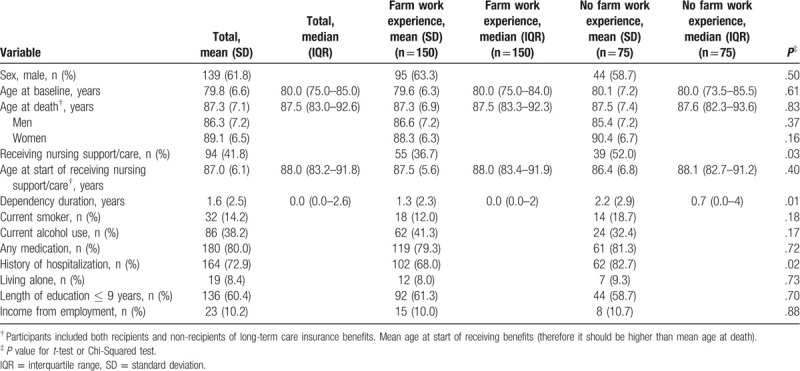
Characteristics at baseline of the 225 participants in the Yamanashi Healthy–Active Life Expectancy cohort study who had died by 2017, stratified by farm work experience.

Table 2 shows the estimated duration of dependency stratified by personal characteristics. Mortality rate, mean lifespan, and the proportion of individuals receiving LTCI in the cohort did not differ significantly between individuals with farm work experience and those without. The duration of dependency was longer for individuals who were older at baseline and shorter for those with farm work experience and those who were not taking any medications at baseline. Table 3 shows the primary results stratified by sex. The duration of dependency was significantly shorter in men with farm work experience than in those without. It was also shorter in women with farm work experience; but the difference was non-significant.

**Table 2:**
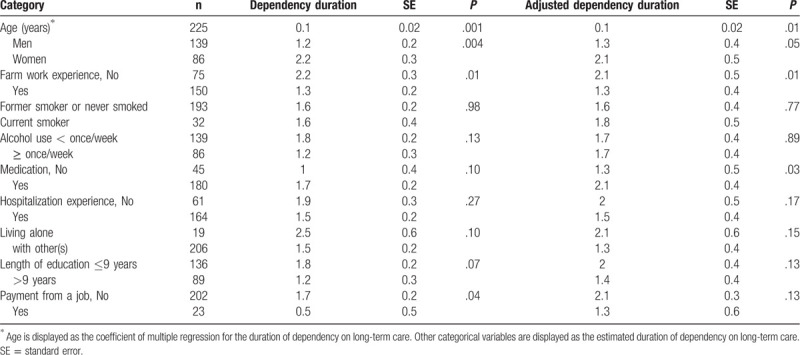
Estimated mean duration of dependency on long-term care among older adults stratified by personal characteristics.

**Table 3:**
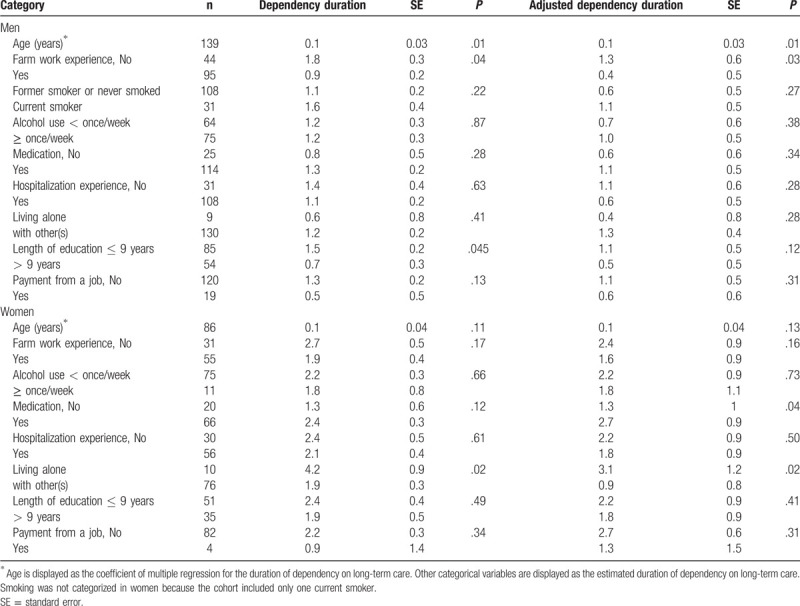
Estimated mean duration of dependency on long-term care stratified by sex.

Table 4 shows the results of the sensitivity analysis after excluding participants who died within the first 2 years of the follow-up period. The duration of dependency was significantly longer for individuals who were older (vs younger) at baseline and significantly shorter in participants with (vs without) farm work experience. A second sensitivity analysis that adjusted for exercise habits and social activities and checked whether participants eat 3 meals a day and consume fruit daily found a shorter duration of dependency in individuals with (vs without) farm work experience (additional details in Supplementary Table 2, http://links.lww.com/MD/E868).

**Table 4:**
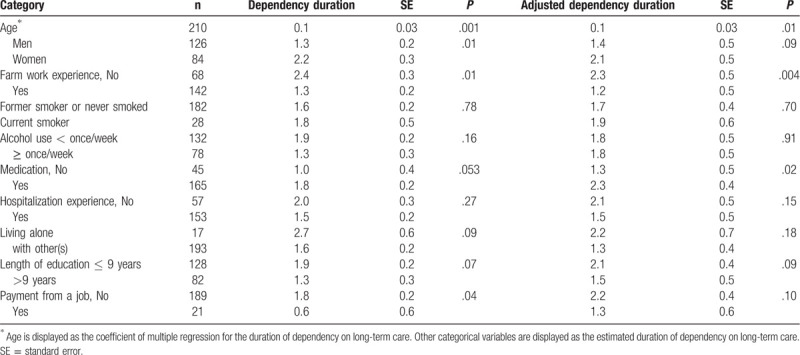
Estimated mean duration of dependency among older adults who died between 2006 and 2017 (sensitivity analysis).

## 4 Discussion

The Japanese cohort data showed that older adults with farm work experience had a significantly shorter dependency duration that did those without said experience. This result was confirmed in a stratified analysis for men, and in a sensitivity analysis of reducing reverse causality.

### 4.1 Results in the context of previous reports

A 2008 Japanese report using data from the LTCI database indicated that the duration of dependency in older men was 1.60 years in the general population and 1.48 years among farmers.^[32]^ In older women, the dependency duration was 3.30 years in the general population and 3.13 years in the Yamanashi region, where our research was conducted. The definition of dependency duration in the previous report was “time for receiving insurance benefits for a care need of 2–5 ranks.” The estimated dependency duration of our study was lower in both men and women with farm work experience as compared to those without. Although the definitions between the previous and our data differed, the results were consistent in that farm work experience reduced dependency duration.

Our data were also consistent in that farm workers had a lower prevalence of current smoking^[5,17–19]^ and were more likely to exercise^[7]^ than were the general population. In our study, the proportion of hospitalization among those who had farm work experience was significantly lower than that among those without. However, the proportions of participants taking medication was similar in both groups. It is possible that farm workers were less likely to be hospitalized simply because they lived further from hospitals.^[24]^

### 4.2 Healthy worker effects

Healthy worker effects need to be considered because agricultural work requires physical demands^[33]^; thus, farm workers may be healthier than other people, which is consistent with our results concerning dependency duration. Specifically, those with farm work experience had a significantly shorter dependency duration than did those without, including in our current sensitivity analysis. In addition, baseline ages were older for farm workers compared to their counterparts. Older people may be at high risk of care need; the baseline age difference strengthens our conclusion.

The mortality rate of the original cohort was 43.2% vs 37.9% (*P* = .22), mean life expectancy was 87.3 years vs 87.5 years (*P* = .83), the proportion receiving LTCI benefits was 30.9% vs 36.5% (*P* = .21), and mean age at first receipt of benefits was 85.2 years vs 85.6 years (*P* = .70). Without equivalence testing, the outcomes for the 2 groups look very similar. Farm work may be associated with other physical and social activities, and these may affect farm workers dependency duration. The healthy worker effect may be eliminated only in a randomized controlled trial; however, an observational study would not completely eliminate the effects. In most cases, the findings from observational studies and randomized controlled trials may be similar,^[34]^ while the results vary with adjustment covariates.^[34,35]^ An observational study could estimate the primary outcome in association with common exposure.^[36]^

### 4.3 Possible explanations for our findings

In older adults, the reasons for requiring nursing support or care include cerebrovascular diseases, fracture, musculoskeletal diseases, and dementia.^[26,27]^ A Japanese longitudinal study suggested that middle-aged and older adults who increased their walking by 30 minutes per day (1.75 metabolic equivalents^[37]^) had a significantly lower risk of needing nursing care through LTCI than did those who did not increase their walking.^[38]^ Studies suggest that exercise load is approximately 3.8 metabolic equivalents for gardening and 4.8 for professional farm work.^[37]^ Farm working is therefore considered likely to decrease the risk of needing nursing care. Our sensitivity analysis controlling for exercise habits and social activities also suggested that farm work would reduce dependency duration; although, the data lacked exercise load from farm work. The data also lacked information concerning vegetable intake amount. Farm workers likely eat more vegetables than do the general population, and eating vegetables is reportedly associated with agricultural work in rural areas.^[7,16]^ Those with farm work experience may have reduced dependency duration due to a healthy diet and exercise.

Previous studies suggested that gardening and other productive activities have a protective effect against dementia among older adults.^[8,9]^ Another study showed the levels of brain-derived neurotrophic factor and platelet derived growth factor were significantly increased after gardening.^[39]^ These studies suggest the potential benefit of distinct activities on cognitive function and memory. Because gardening may increase brain-derived neurotrophic factor and platelet derived growth factor,^[39]^ the incidence rate of dementia may decrease.

### 4.4 Implications for policymakers

Policymakers could refer to the present results to save medical cost expenditure by encouraging older residents to actively work on a farm. The shorter dependency duration in those with farm work experience is attributed to 3 reasons: farm workers do not retire early and work to a very old age^[40]^; older farmers still enjoy being productive, which contributes to good mental health^[41–43]^; and continuing to work to an old age ensures that they maintain social capital and additional financial security.^[44]^ Of the 47 prefectures in Japan, those with high agricultural employment rates in adults aged 65 years or older have lower medical cost expenditures related to older adults.^[45]^ Policymakers focusing on preventing the need for nursing care in older populations should consider promoting farming or gardening. People who own land can easily understand and take part in simple agricultural practices.

### 4.5 Limitations and strengths

This study had several limitations. First, some information was missing, such as BMI and nutrition status, which was absent in the baseline survey, and so could not be included in the analysis. Farm workers may have been physically active^[5–7]^ and had high vegetable intake,^[7,16]^ which may have affected their BMI. The control group may have included more unhealthy people and may have a longer estimated duration of dependency. Information about duration of farm work experience was almost missing. We classified individuals according to whether they were engaged in farm work or not at the baseline survey, not according to the duration of the experiences. Therefore, it is possible that participants who became older and retired from farm work were classified into the non-experience group. Second, only 33.0% women (86 women) died during the approximately 13-year follow-up period. This resulted in lower power for the stratified results for women. Third, the investigation was conducted in a single prefecture of Japan; thus, our results cannot be generalized to the entire Japanese population.

This study also had several strengths. First, although researchers have addressed mortality among farm workers, there is less evidence concerning dependency duration. Second, the results were from a cohort with semi-random sampling from a prefecture comparable to the Japanese general population. Third, the follow-up duration was around 13 years, and the follow-up rate was high (91.3%). This enabled us to thorough examine the interval between nursing support or care and death. Few previous cohort studies have measured both of these outcomes.

The duration of dependency on nursing care tends to be longer globally. Making people live active and healthier lives may be meaningful to individuals and may reduce medical costs for the country. Further research is needed on how the farm work experience is related to the duration of dependency and is also applicable to a diverse population of people with different crops and cultivated areas around the world.

## 5 Conclusion

The investigation of Japanese community-dwelling people suggested that farm work was significantly associated with short dependency duration in late life. This association was more marked in men than women. Farm work may therefore contribute to reducing dependency duration and decrease the cost of nursing care.

## Acknowledgments

We express our gratitude to all participants in the Y–HALE cohort and to all public officers in Yamanashi Prefecture. Special thanks are also extended to the past and current staff members of the Department of Health Sciences, University of Yamanashi, for their contributions and cooperation in launching and running this cohort study. We also thank Editage (www.editage.com) for English language editing.

## Author contributions

**Conceptualization:** Kayo Haruyama, Zentaro Yamagata.

**Data curation:** Kayo Haruyama.

**Formal analysis:** Kayo Haruyama.

**Funding acquisition:** Hiroshi Yokomichi.

**Investigation:** Hiroshi Yokomichi, Zentaro Yamagata.

**Methodology:** Kayo Haruyama, Hiroshi Yokomichi, Zentaro Yamagata.

**Project administration:** Kayo Haruyama, Hiroshi Yokomichi, Zentaro Yamagata.

**Resources:** Hiroshi Yokomichi, Zentaro Yamagata.

**Supervision:** Hiroshi Yokomichi, Zentaro Yamagata.

**Validation:** Hiroshi Yokomichi, Zentaro Yamagata.

**Visualization:** Kayo Haruyama, Hiroshi Yokomichi, Zentaro Yamagata.

**Writing – original draft:** Kayo Haruyama, Hiroshi Yokomichi.

**Writing – review & editing:** Hiroshi Yokomichi, Zentaro Yamagata.

## Abbreviations

Abbreviations: LTCI = long-term care insurance, SD = standard deviation, SE = standard error, Y–HALE = Yamanashi Healthy–Active Life Expectancy.
